# Can Physiological Endpoints Improve the Sensitivity of Assays with Plants in the Risk Assessment of Contaminated Soils?

**DOI:** 10.1371/journal.pone.0059748

**Published:** 2013-04-02

**Authors:** Ana Gavina, Sara C. Antunes, Glória Pinto, Maria Teresa Claro, Conceição Santos, Fernando Gonçalves, Ruth Pereira

**Affiliations:** 1 Departamento de Biologia, Universidade de Aveiro, Campus de Santiago, Aveiro, Portugal; 2 CESAM – Centro de Estudos do Ambiente e do Mar, Universidade de Aveiro, Campus de Santiago, Aveiro, Portugal; 3 Departamento de Biologia da Faculdade de Ciências, Universidade do Porto, Rua do Campo Alegre, Porto, Portugal; Dowling College, United States of America

## Abstract

Site-specific risk assessment of contaminated areas indicates prior areas for intervention, and provides helpful information for risk managers. This study was conducted in the Ervedosa mine area (Bragança, Portugal), where both underground and open pit exploration of tin and arsenic minerals were performed for about one century (1857 – 1969). We aimed at obtaining ecotoxicological information with terrestrial and aquatic plant species to integrate in the risk assessment of this mine area. Further we also intended to evaluate if the assessment of other parameters, in standard assays with terrestrial plants, can improve the identification of phytotoxic soils. For this purpose, soil samples were collected on 16 sampling sites distributed along four transects, defined within the mine area, and in one reference site. General soil physical and chemical parameters, total and extractable metal contents were analyzed. Assays were performed for soil elutriates and for the whole soil matrix following standard guidelines for growth inhibition assay with *Lemna minor* and emergence and seedling growth assay with *Zea mays*. At the end of the *Z. mays* assay, relative water content, membrane permeability, leaf area, content of photosynthetic pigments (chlorophylls and carotenoids), malondialdehyde levels, proline content, and chlorophyll fluorescence (F_v_/F_m_ and Φ_PSII_) parameters were evaluated. In general, the soils near the exploration area revealed high levels of Al, Mn, Fe and Cu. Almost all the soils from transepts C, D and F presented total concentrations of arsenic well above soils screening benchmark values available. Elutriates of several soils from sampling sites near the exploration and ore treatment areas were toxic to *L. minor*, suggesting that the retention function of these soils was seriously compromised. In *Z. mays* assay, plant performance parameters (other than those recommended by standard protocols), allowed the identification of more phytotoxic soils. The results suggest that these parameters could improve the sensitivity of the standard assays.

## Introduction

Plants are essential components of ecosystems as they are primary producers of organic matter and oxygen, and a food source for heterotrophic organisms, humans included. They are considered versatile tools to monitor the presence and the effects of pollutants in soil, for they are in close contact with the soil matrix and with soil pore water, absorbing both nutrients and pollutants and responding to changes in soil properties [1], [2], [3], [4]. Several are the reasons why plants have been widely used in assays, to evaluate soil quality and risk assessment of phytotoxic compounds: i) they have a sedentary existence, so they can be continuously exposed to a source of pollution throughout their life cycle; ii) seeds are relatively inexpensive and plants are easily cultured in laboratory; iii) their biological responses can be evaluated in a short period of time and, iv) their condition/performance can be monitored in different ways, from physical observations to spectroscopic methods [5], [6], [7]. In order to ensure comparability of results across studies and laboratories, there is a list of standardized plant species that can be used in toxicity tests [8], [9].

As far as tests with terrestrial plants are considered, the standardized protocols suggest that parameters such as seed germination, growth above soil and/or root growth have to be evaluated [8], [10]. As with other tests, these can be considered acute when they evaluate potential immediate effects, as inhibition of seed germination, inhibition of seedling growth and biomass production, and chronic when evaluating long-term effects involving those occurring in the life cycle of the plant [11]. However, there are several other ecophysiological parameters that can be evaluated in plants, which can potentially be more sensitive and indicative of stress conditions. These parameters are usually not considered in plant tests because they are not previewed in standard protocols. However, besides the standard parameters, the evaluation of other physiological (e.g. chlorophyll fluorescence, pigments content) and biochemical (e.g. content of malondialdehyde and proline, enzymes activity) parameters may also be important [2], [6], as they can help finding out potential false negative results.

Photosynthesis is a core function in the physiology of plants, during which light is captured by chlorophyll molecules and by two photosystems (PSI and PSII) in the membrane of thylakoids and then used to remove electrons from water molecules. Such electrons are transported through an electron transport system and finally accepted by NADP^+^ molecules. Meanwhile, the transportation of electrons occurs in close association with the passive movement of protons to the lumen of thylakoids. The energy of this gradient is used for the phosphorylation of ADP. Both ATP and NADPH molecules are key products for CO_2_ fixation and the production of sugars in the dark step of the process (Calvin Cycle) [12].

The photosynthetic system of higher plants has been shown to be sensitive, reacting to different kinds of stress agents like drought [13], salinity [14], metals [15], [16], [17] and herbicides [18], in shorter periods of time. During stress conditions plants lose their ability to use light energy and dissipation mechanisms are triggered to protect the plant from photoinhibition and photoxidation [19]. The excess of light energy can be dissipated as heat or as chlorophyll fluorescence [20]. Hence, impairments in the photosynthetic activity can be evaluated measuring chlorophyll fluorescence parameters like Φ_PSII_, which measures the efficiency of the PSII photochemistry (i.e. the proportion of light absorbed by chlorophyll molecules used in photochemistry reactions) and F_v_/F_m_ the maximum efficiency of PSII (the efficiency of the PSII when all the reactive centres are open) [20]. The evaluation of these parameters has been facilitated by the marketing of user friendly and portable devices, which makes routine evaluations possible. Further, these measurements have the great advantage of being non-destructive allowing multiple evaluations throughout plant exposures to stressful conditions.

Additionally, when the rate of excitation of chlorophyll molecules exceeds the conversion of energy in the reaction centres of PSII, excited chlorophyll molecules can generate singlet oxygen molecules, which can promote photoxidation. At this stage, carotenoids, which are also components of PSII, take action, as non-enzymatic antioxidants, scavenging excited chlorophyll molecules and dissipating energy as heat [14], [19]. However, not only singlet oxygen species but also other reactive oxygen species (ROS), generated by different stress agents, may induce oxidative damage to pigments, impairing overall photosynthetic activity (photoinhibition). The aminoacids metabolism has being shown as crucial in the response of plants to oxidative stress agents because aminoacids like proline, amongst other functions, may act as hydroxyl radical scavengers [21].

Having in mind all of these mechanisms involved in plants response to toxicants, the aim of the present study was to evaluate the ability of new endpoints to increase the sensitivity of plant assays, to identify natural soils, seriously contaminated with metals, based on their phytotoxicity. To attain this purpose, both the whole soil matrix and soil elutriates, for a set of soil samples from an abandoned mine area, were assessed through seed germination and growth assay with *Zea mays* and a growth inhibition assay with *Lemna minor*, respectively. The assays were performed according to, standard protocols. Further, other plant physiological parameters as water content, chlorophylls (*a* and *b*) and carotenoids content, chlorophyll fluorescence (F_v_/F_m_ and Φ_PSII_), membrane permeability and oxidative stress parameters (proline and MDA content) were assessed in *Zea mays* at the end of the assay. Here, we hypothesized that more soils will be identified as phytotoxic, if more plant performance parameters are measured. Here we hypothesized that the more plant performance parameters are measured, the more soils will be identified as phytotoxic.

## Materials and Methods

No specific permisiions were required for these locations activities. We confirm that the location is not privately-owned or protected in any way and we confirm that the field studies did not involve endangered or protected species.

### Study site and soil sampling

**Figure 1:**
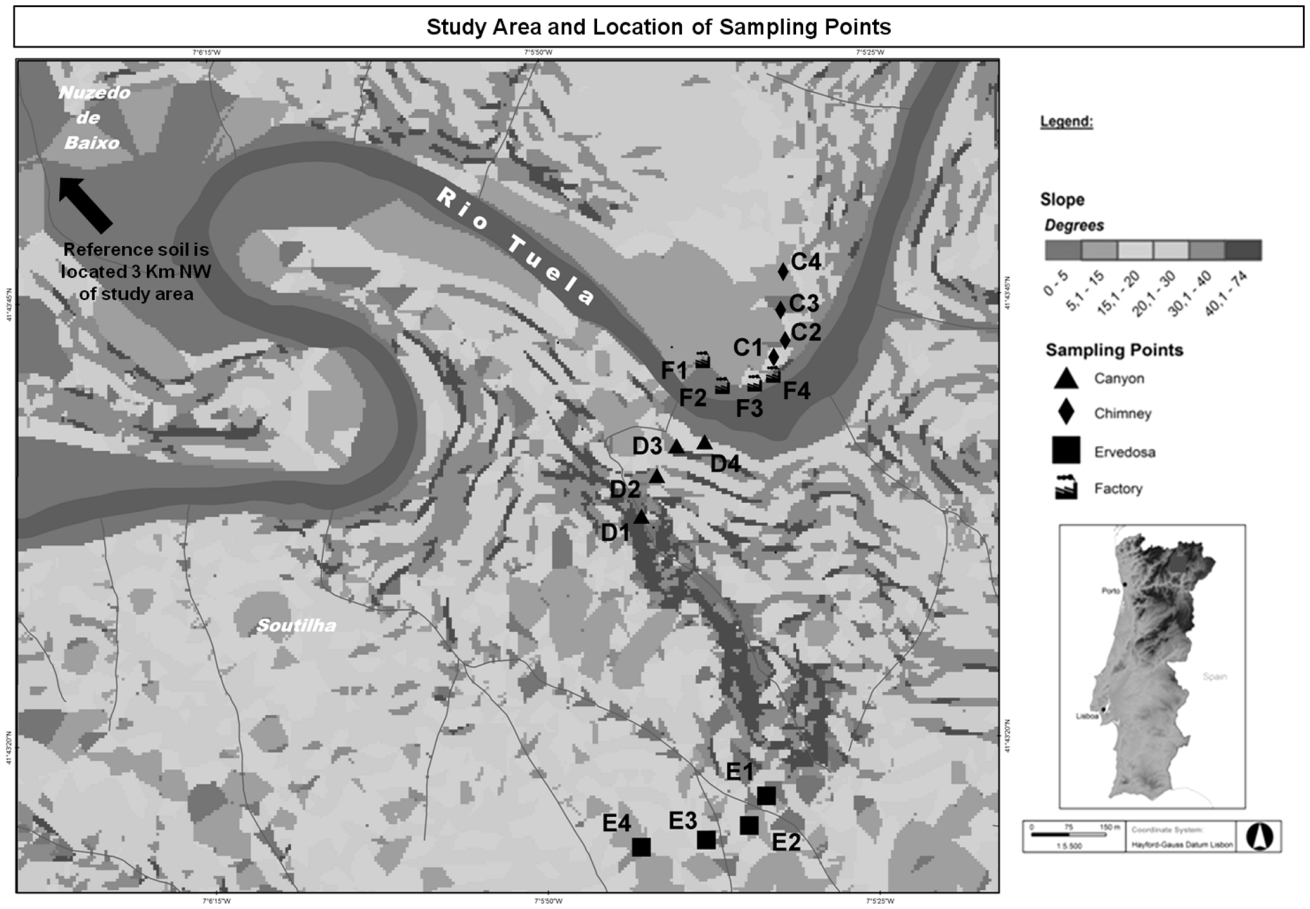
Study area and location of transects and sampling points (adapted from Carvalho et al. [**22**]).

The Ervedosa Mine is located in Vinhais, district of Bragança, in northeast Portugal. In this mine arsenic (As) and tin (Sn) were explored for about one hundred years (1857–1969) (figure 1) deeply changing the overall landscape [22]. Environmental contamination of local soils by metals was evaluated and reported by Novais [23]. The levels of metals detected in soils, of this area, have raised concerns about the potential risks to local natural communities. Some soils have also shown to be highly toxic for species like *Eisenia andrei*, *Folsomia candida*, *Pseudokirchneriella subcapitata*, *Daphnia magna* and *Vibrio fischeri* (unpublished data) confirming their hazard for edaphic species.

In the mine area four transects (C, D, E and F) were considered with four sampling points each, set apart from each other for about 50 m (figure 1). Additionally, a reference site was selected, 3 km away from the mine area. Transect C began in the ore treatment area and extended north. Transect D extended from the mining area to the river Tuela. Transect E started in the ore exploration area and extended south, to the Ervedosa village. Further, transect F was set parallell to the river Tuela and crossed the area where the ore was treated to extract metals of interest.

Surface soil samples (0–20 cm) were collected in the seventeen sampling points and brought to the laboratory where they were left to dry at room temperature. Thereafter the samples were sieved and the <4 mm fraction was stored for physical and chemical characterization and for plant assays.

### General physical and chemical characterization of soil samples

Soil conductivity was measured in a soil-water suspension according to the method described by FAOUN [24]. For this purpose 10 g of soil, were mechanically shaken with 50 mL of distilled water during 15 min. The suspension was left to rest overnight and conductivity was measured using a pre-calibrated LF330/SET conductivity meter. Soil pH_KCl_ was measured in a suspension of soil, prepared with a solution of KCl 1M, according to ISO 10390 [25].

Water holding capacity (WHC) of soils was measured according to the procedure described in the ISO 10390 guidelines [25]. Soil samples were placed in polypropylene flasks, with the bottom replaced by filter paper and immersed in water for 3 h. After this period flasks were placed on absorbent paper for 2 h to reject the excess of water that could not be retained by soil. The WHC was then determined by weighting each replicate before and after drying at 105°C until weight stabilization [25].

Soil water content (moisture) was determined by weight loss, at 105°C, for 24 h. The organic matter content (OM) was determined by weight loss on ignition at 450°C, during 8 h, according to SPAC [26]. All parameters described above were measured in three soil replicates.

### Soil metal content: total and extractable concentrations

The content in metals of soil samples was determined by two extraction methods: a strong one with *aqua regia* and a mild extraction with calcium chloride 0.01 M [27]. For the *aqua regia* extraction, 1 g of each soil replicate was digested with 3 mL of 37% hydrochloric acid (*pro analysis*, Panreac) and 1 mL of 65% nitric acid (Suprapur, Merck), in closed Teflon flasks. The flasks were heated on a sand bath at 100°C for 5 h. After this period, 10 mL of HNO_3_ (4N) were added to the flasks and the solution was filtered, through 0.2 µm FT30/0.2CA-S filters, to remove all coarser particles, and transferred to polypropylene volumetric tubes. At the end of acid digestion the volume of each extract was adjusted with distilled water until a final volume of 25 mL was attained. For quality control of the extraction procedure, the same process was carried out using the same reagents but without the soil sample and three blank samples were prepared and sent for analysis. For the extraction with calcium chloride 0.01 M, suspensions of soil in the CaCl_2_ solution (0.01 M) (1∶10 m/v)) [27] were prepared for all the soil replicates. The soil suspensions were shaken mechanically for 2 h, at 20±2°C. After this, the suspensions were centrifuged at 4000 rpm and stored (acidified to pH<2 with HNO_3_) for quantification of metals. Total and extractable concentrations of Al, Pb, P, V, Mn, Fe, Cu, Zn, As, Sb, Ba and Sn were analyzed by ICP-MS (Thermo X-Series quadrupole ICP-MS, Thermo Scientific).

### *Lemna minor* assay

*L. minor* was obtained from laboratorial cultures reared under controlled conditions (temperature 20±2°C; photoperiod 16 h^L^:8 h^D^; illuminance: 10000 lux) in Steinberg medium according to the guideline OECD 221 [28]. The tests were performed with soil elutriates obtained from suspensions of soil samples in Steinberg medium (1∶4 m/v). These suspensions were mechanically shaken overnight and then left to stand for 12 h for sedimentation. After this period, suspensions/elutriates were decanted and the supernatant portion was collected. *L. minor* was exposed, in three replicates, to a range of elutriate dilutions (100 mL/replicate). The assay was started placing nine fronds of *L. minor,* per vessel, under the controlled conditions described above. In the control replicates *L. minor* fronds were exposed only to the Steinberg medium. After 7 days of exposure, the fronds of each replicate were collected, dried at 70°C, till weight stabilization, and weighted. Growth rate was quantified according to the equation: GR = (Ln (W_f_)-Ln(W_i_))/7 (W_f_ and W_i_ are final and initial weights, respectively) [28]. IC_50_ values and corresponding 95% confidence limits, for each elutriate, were determined by nonlinear regression analysis, fitting a logistic equation to the data using technique of least squares. The software Statistica 10.0 was used for this purpose.

### *Zea mays* seed germination and growth assay

Seed germination and growth assay with *Z. mays* were performed according to the ISO 11269-2 guideline [8]. Seeds were purchased from a local supplier and the damaged ones were discarded after visual inspection. Assays were performed in plastic pots, which were filled with 200 g of soil (four replicates per soil). Control was conducted with OECD standard soil [29]. Twenty seeds were added to each pot. In the beginning of the assay, a commercial solution of nutrients (Substral^TM^ 10%) was added to each pot. Pots were maintained at controlled temperature (20±2°C), photoperiod (16 h^L^: 8 h^D^) and illuminance (about 25000 lux). During daily observations, the number of emerged seeds was recorded and the water content of the pots was checked and adjusted. Only the first five emerged seeds were left to growth, the remaining ones were counted and harvested. The assay was validated and started after 50% of the seeds from the control pots emerged. Fourteen days later, the assay finished. Chlorophyll fluorescence measures were taken in the adaxial side of leaves of two plants from each soil replicate. The biomass above soil was harvested (only for four plants per pot) and wet weight was immediately determined. Dry biomass was weighted after drying at 70°C.

The leaves collected to measure water content and membrane permeability of plant cells were immediately processed, while the leaves for the quantification of chlorophylls, carotenoids, proline and malondialdehyde content were immediately frozen in liquid nitrogen and stored in a deep freezer for further analysis. These parameters were measured in plant leaves that were collected from one plant per replicate.

### Plant performance parameters in *Zea mays* assay

#### Specific leaf area

The leaves harvested to determine specific leaf area (SLA) were placed on graph paper (used as scale) and then photographed. Afterwards the leaf area was determined with the ImageJ 1.43 µ software (Internet free). The leaves were weighed on an analytical balance and were dried at 60°C, until stabilization, and the dry weight was determined. SLA was then calculated as the ratio of leaf area (cm^2^) to leaf dry weight (g).

#### Photosynthetic pigments (chlorophyll and carotenoid contents)

Chlorophylls (chl *a* and chl *b*) and carotenoids were determined spectrophotometrically according to the method described by Sims and Gamon [30]. Pigments were extracted from leaves samples of about 0.5 g, and were homogenized in 2 mL of cold acetone (99% Cleanse®)/Tris buffer 50 mM (99.8% Merck®) (80∶20, v/v). Then the extracts were transferred to centrifuge tubes, homogenized in vortex for about 30 s and centrifuged for 5 min, at 4000 rpm and 4°C. The supernatant was transferred to new tubes which were stored in ice and in the dark. The extraction procedure was repeated by adding more 1.5 mL of the same extraction solution to the pellet. The resulting supernatant was collected into former tubes, kept in the dark, and once again the extraction solution was added till a final volume of 6 mL was attained.

The quantification of chlorophyll (*a* and *b*) and carotenoid contents was achieved by spectrophotometry, measuring absorbance of the extracts at 470, 537, 647 and 663 nm in a Thermo Scientific Vis Spectrophotometer 10S TM. The extraction solution was used as blank for zeroing the absorbance.

#### Malondialdehyde content

The content of malondialdehyde (MDA) in samples of plant tissue was determined by the thiobarbituric acid method as described by Elkahoui et al. [31]. The MDA is an end product of lipid peroxidation in plant cells. Hence samples of leaves, of about 0.5 g, from one plant per replicate, were homogenized with 5 mL of 0.1% trichloroacetic acid (TCA) (Riedel-de Haën). The homogenates were centrifuged for 5 min, at 4000 rpm and at 4°C. Then, aliquots of 1 mL of the supernatant were transferred to falcon tubes and 4 mL of 20% TCA solution containing 0.5% of thiobarbituric acid (TBA) (≥98%, Sigma-Aldrich) were added to the tubes. The tubes were placed in a water bath, at 95°C, for 30 min. After cooling in ice, the tubes were centrifuged for 10 min at 4000 rpm and at 4 °C. The specific and the non-specific absorbance of the supernatant were measured at 532 and 600 nm, respectively. Distilled water was used as blank for zeroing the absorbance of the spectophotometer Thermo Scientific TM 10S Vis. The MDA content was calculated subtracting the non-specific absorbance at 600 nm and using the molar extinction coefficient ε = 155 mM^−1^ cm^−1^.

#### Proline content

The proline content of plant leaves was determined according to the method described by Khedr et al. [32]. From each plant (one plant per replicate) about 100 mg of leaves were homogenized in 1.5 mL of 3% sulfosalicylic acid (≥99%, Sigma). After centrifugation of the extracts at 4000 rpm, 100 µL of the supernatant were transferred to new tubes and mixed with 2 mL of glacial acetic acid (pro analysis, Panreac) and 2 mL of ninhydrin (Riedel-de Haën). The mixture was incubated in a water bathat 100°C, for 1 h. After this period, the tubes were placed in ice, and 1 mL of toluene (99.9%, Merck) was added to cooled tubes, in a hote. Absorbance of the chromophore solution was measured at 520 nm in a Thermo Scientific TM 10S Vis spectrophotometer [32]. The content of proline in samples was then extrapolated from a calibration line obtained measuring the absorbances of proline solutions of known concentration (0.2, 0.1, 0.05, 0.025, 0.0125 mg mL^−1^).

#### Relative water content

For the evaluation of this parameter each leaf was weighed on an analytical balance (F_W_), and then placed in a Falcon tube completelly filled with distilled water. The tubes were left in the dark, at 4°C, for 12 h. After this periodthe leaves were removed from water and placed on an absorbent paper to remove the excess of water, and the turgid weight (T_w_) was determined. Afterwards, leaves were dried at 60°C, until stabilization and the dry weight was determined (D_w_). The relative water content of plant leaves (RWC) was calculated using the following equation, and expressed as a percentage:
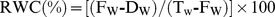



#### Chlorophyll fluorescence (Fv/Fm and ΦPSII)

Chlorophyll fluorescence measurements were performed on the same expanded leaves of each plant using a portable fluorometer (Minipan Photosynthesis Yield Analyser, Walz, Effeltrich, Germany). Light exclusion clips were placed on the adaxial side of the leaves for 30 min and the following chlorophyll (chl) fluorescence measurements were taken [20]: minimum chl fluorescence in the dark adapted state (F_0_), when all the reaction centres of PSII are opened; maximum chl fluorescence in the dark adapted state (F_m_), after a pulse of actinic light (0.8 s to 8000 micromol m^−2^ s^−1^) has closed all the reaction centres of PSII; the steady state chl fluorescence in the light adapted state (F_t_); and the maximum chl fluorescence in the light adapted state (F'_m_) after the same pulse of actinic light has been applied. With these measurements the efficiency of photosystem II (quantum yield) (Φ_PSII_) and the maximum quantum yield or the maximum photosynthetic efficiency of photosystem II (F_v_/F_m_) were calculated based on the following equations:
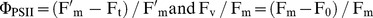



#### Membrane permeability

Membrane stability was estimated indirectly through quantification of electrolyte leakage according to, the method described by Lutts et al. [33]. One leaf from each replicate was weighed, washed with Milli Q water and then placed in falcon tubes filled with Milli Q water. The ratio mass/volume was the same in all the tubes. The tubes were shaken mechanically for 12 h in an orbital shaker. At the end of this period, the conductivity of the solution was measured with a conductivimeter (CONSORT C830 - Multi-parameter analyzer) (C_inicial_). Then the vials were placed in the autoclave for 10 min, at 121°C. After cooling, the conductivity of the solution (C_final_) was measured again. The membrane permeability and the ratio of conductivities C_inicial_/C_final_ were calculated and expressed as a percentage.

### Stastical analysis

To test for significant differences in the parameters measured in plants, exposed to different mine soils, one-way analysis of variance (ANOVA) was performed, after the Levene's test for checking homogeneity of variances. When significant differences were recorded by the one-way analysis of variances, a two-tailed Dunnet or Games-Howel test (GHT) (when the assumption of equal variances was not accomplished) was perfomed to compare each soil with the REF soil, in terms of the paremeter under evaluation. The authors chose parametric tests, instead of non-parametric tests, even when the assumptions were not met, because one–way ANOVA has proved to be robust even when some deviations from requirements occur [34].

## Results and Discussion

Soil contamination is considered one of the main causes of soil degradation worldwide and in Europe in particular [35]. After the recognition of the high rate of the verified soil loss, the European Union has developed new legal documents to protect the soils within the European territory. Within this scenario a soil framework directive was proposed and has been under discussion, since 2006 [36]. Amongst other aspects, this directive states that each member state should provide a list of the contaminated sites within their territory [36]. Such requirement will lead all the member states to enforce the application of environmental risk assessment (ERA) frameworks. Phytotoxic tests are required by ERA frameworks [37] to assess soil habitat, retention (aquatic species) and production functions. Bearing this idea in mind, this work was developed to assess the phytotoxicity of soils collected in the Ervedosa mine (north of Portugal) explored in the past for tin and arsenic. Further, we have hypothesized that we can improve the sensitivity of the standard phytotoxic assays evaluating other plant physiological, biochemical and chlorophyll fluorescence parameters, based on the assumption that these parameters will be able to detect stress before visible signs have evolved.

### Soils physical and chemical characterization

The average values recorded for the different physical and chemical parameters measured in each soil sample collected in the Ervedosa mine area are described in table 1. In general, the soils had low pH (below 4.6±0.02 recorded in the REF soil) as well as low conductivity values. Soil F1 displayed the lowest pH_KCl_ (3.4) and the highest conductivity value (290.33 µS cm^−1^). Regarding the content of organic matter (OM), and according to USEPA [38] classification, soils were grouped into: i) low content (<2%) – soils D2, E1and F3; ii) medium content (2%≤OM<6%) – soils REF, C1, C3, D1, D3, D4, E2, F1 and F2 soils and iii) high content (≥6%) – soils C2, C4, E3, E4 and F4. Soil E4 presented the highest organic matter content (19.7%) as well as the highest water holding capacity (84.6%).

**Table 1:** General physical and chemical parameters measured in soil samples collected in the Ervedosa mine area (average ± STDEV): pH_KCl_, conductivity, MO -organic matter (%) and WHC_max_ – maximum water holding capacity (%).

	pH_KCl_	Conductivity(µS cm^−1^)	OM (%)	WRC (%)
REF	4.6±0.02	51.1±0.76	3.8±0.4	22.5±0.1
C1	3.8±0.02	35.7±0.41	4.4±0.4	38.6±0.6
C2	4.6±0.66	36.7±0.5	9.8±0.3	57.7±0.5
C3	4.0±0.01	25.8±1.34	5.6±0.3	40.1±2.3
C4	4.1±0.01	35.1±0.31	7.7±0.2	52.8±0.9
D1	4.0±0.01	34.0±4.40	4.4±0.0	62.5±12.5
D2	4.4±0.01	19.5±3.64	1.3±0.1	8.9±0.1
D3	4.0±0.02	41.0±2.77	2.4±0.3	35.4±0.3
D4	4.4±0.02	12.2±0.41	4.9±0.1	23.3±0.1
E1	4.3 ±0.01	7.5±0.07	1.6±0.5	34.3±2.4
E2	4.3±0.00	20.0±0.29	5.4±0.3	40.8±1.8
E3	3.8±0.04	22.8±0.98	10.9±0.4	66.3±0.9
E4	3.8±0.01	40.0±7.76	19.7±0.3	84.6±2.1
F1	3.4±0.04	290.3±7.75	2.0±0.1	27.9±1.0
F2	3.6±0.05	54.3±12.81	3.0±0.3	33.1±3.9
F3	4.0±0.01	16.3±0.33	1.5±0.1	31.8±0.4
F4	4.1±0.01	32.3±0.67	6.7±0.2	46.8±3.4

Highest values recorded for each parameter were highlighted with bold letter.

### Total and extractable metal concentrations

Generally, the highest total concentrations of metals were recorded in soils from transects D, E and F. Except for Al, Mn and Fe, which were recorded in high concentrations in all the soils, including the REF soil. When compared with some soil benchmark values available, almost all the soils from transepts C, D and F presented concentrations of arsenic well above the EPA ECO-SSL (18 mg kg^−1^) (a plants soil screening benchmark) (http://rais.ornl.gov/) as well as above the HC_5_ value proposed for this metalloid (5.63 mg kg^−1^) by Jänsch et al. [39]. The HC_5_ values proposed by these authors were calculated based on EC_50_ values obtained for different species of animals, plants and microbial processes in chronic tests. These results represent the concentrations of the metals below which no more than 5% of the species and/or microbial processes will show a detrimental effect of 50%. This observation creates suspicions about the potential phytoxicity of almost all the soils analyzed in this study, since benchmark values for As were clearly surpassed, as previously mentioned. However, it seemed that this element was particularly available for plants especially in the soil F1, which showed the highest concentration of As in calcium chloride extracts (Table 2). The same calcium chloride extract obtained for soil D1, showed the highest concentrations of P, Mn, Fe and Cu. In fact all the soils from transect D and soil E1 had total concentrations of Cu, well above the EPA ECO-SSL (70 mg kg^−1^)) and the C_5_ value (55 mg kg^−1^) proposed by Jänsch et al. [39]. Soils D2 and F2 had total concentrations of lead also above the soil screening benchmarks mentioned (EPA Eco-SSL: 120 mg kg^−1^; HC_5_: 163.5 mg kg^−1^), and the same was observed in terms of the total concentration of zinc in soils D1, E1 and E4. Nevertheless, in all the other soils (except D1 and F1), the extractable concentrations of metals were not meaningful, except for Al. The lack of correlation between soil total metal contents and the levels bioaccumulated by plants has been pointed out by several authors. Subsequently, the use of neutral salt solutions has been recommended based on the assumption that the cations provided by these salts are able to displace metals located on mineral surfaces, to the aqueous phase [40], mimicking processes occurring in rhizosphere microenvironment. In fact plants can make metal ions more available in the rhizosphere, both increasing acidity with the support of proton pumps localized in their plasma membranes and through the active secretion of low-molecular mass compounds that function as metal chelators [41]. The negative potential of plasma membranes, the existence of Fe^2+^, Ca^2+^, and Zn^2+^ transporter channels of low specificity and of intracellular binding sites for metals are additional driving forces for metals uptake [41] and together they could explain the toxicity of soils other than those with high extractable concentrations of metals.

**Table 2:** Average concentrations (± STDEV) of metals in soils samples collected in the Ervedosa mine area, after calcium chloride (0.01 M) and aqua regia extraction (total metal contents).

	Al	Pb	P	V	Mn	Fe	Cu	Zn	As	Sb	Ba	Sn
*Aqua regia* extraction (µg g^−1^)												
REF	14886.3	27.6	208.6	24.3	132.8	13722.7	26.0	37.4	61.3	0.3	35.8	1.8
C1	9700.0	26.3	199.8	13.5	114.8	22884.8	14.2	46.2	158.7	3.8	19.7	2.3
C2	1963.1	2.5	30.9	bdl	12.9	1152.7	1.2	bdl	0.1	0.1	4.2	0.7
C3	6943.8	21.4	304.0	8.6	53.6	18795.6	11.3	17.6	33.9	2.4	23.2	1.2
C4	865.2	1.2	16.2	bdl	5.8	628.8	1.1	bdl	bdl	bdl	1.7	0.4
D1	4281.7	34.8	4604.0	0.8	1448.3	33536.0	604.8	243.1	3163.5	2.5	13.7	2.5
D2	5056.5	215.7	1344.2	42.5	92.6	16047.8	140.2	68.6	867.6	2.5	31.2	5.5
D3	6916.2	50.2	753.0	36.1	199.6	17220.8	67.0	52.6	323.5	2.6	12.6	2.9
D4	17825.2	30.1	438.8	31.4	538.5	29942.0	128.8	93.5	255.2	2.6	30.6	3.3
E1	12777.5	20.4	223.4	47.6	735.5	25414.9	477.7	232.5	439.4	0.8	27.7	1.5
E2	13752.6	33.9	194.5	22.3	139.6	22676.5	28.6	58.1	68.9	0.8	32.2	3.3
E3	764.1	1.5	31.9	bdl	5.4	1334.4	1.1	bdl	0.3	bdl	1.8	bdl
E4	9675.9	82.7	1227.8	17.2	333.3	45944.2	44.3	461.5	367.4	7.4	97.6	16.2
F1	1084.7	79.2	323.5	bdl	18.0	8348.6	7.9	4.7	15251.8	66.0	61.2	69.7
F2	4044.7	144.7	267.8	17.1	37.2	22555.8	23.0	19.0	13742.9	195.4	46.8	82.1
F3	2099.4	56.0	299.1	1.7	17.7	9634.3	45.2	27.0	7969.1	32.4	30.8	32.7
F4	8754.2	27.4	231.3	12.9	208.7	18965.6	18.2	37.0	47.3	0.8	24.4	1.7
CaCl_2_ extraction (mg L^−1^)												
REF	0.70	0.00	0.01	bdl	0.15	0.06	0.00	0.04	0.00	bdl	0.17	bdl
C1	2.55	0.01	0.02	bdl	0.73	0.12	0.00	0.06	0.01	bdl	0.03	bdl
C2	1.63	0.01	0.01	bdl	3.07	0.12	0.00	0.09	0.00	bdl	0.14	bdl
C3	2.49	0.01	0.01	bdl	0.26	0.08	0.00	0.03	0.00	bdl	0.06	bdl
C4	3.19	0.01	0.02	bdl	0.66	0.22	0.01	0.03	0.00	bdl	0.09	bdl
D1	1.38	0.00	0.20	bdl	3.53	0.60	3.11	1.10	0.09	bdl	0.01	bdl
D2	1.50	0.00	0.04	bdl	0.44	0.09	0.11	0.07	0.02	bdl	0.01	bdl
D3	2.72	0.00	0.02	bdl	0.50	0.18	0.08	0.15	0.01	bdl	0.01	bdl
D4	2.10	0.00	0.05	bdl	0.60	0.33	0.18	0.19	0.01	bdl	0.06	bdl
E1	2.63	0.00	BDL	bdl	0.24	0.04	0.55	0.29	0.00	bdl	0.06	bdl
E2	1.32	0.01	0.01	bdl	0.47	0.13	0.00	0.04	0.00	bdl	0.06	bdl
E3	1.46	0.01	BDL	bdl	1.14	0.15	0.01	0.04	0.00	bdl	0.06	bdl
E4	1.06	0.00	BDL	bdl	0.59	0.25	0.00	0.08	0.00	bdl	0.11	bdl
F1	0.81	0.01	0.06	bdl	0.04	0.07	0.01	0.01	77.49	bdl	0.25	bdl
F2	1.51	0.00	BDL	bdl	0.16	0.14	0.01	0.03	0.32	bdl	0.03	bdl
F3	0.64	0.00	0.01	bdl	0.11	0.10	0.01	0.05	0.27	bdl	0.01	bdl
F4	0.63	0.01	0.17	bdl	0.60	0.42	0.01	1.89	0.03	bdl	0.26	bdl

Highest concentrations were highlighted with bold letter. BDL stands for below detection limit.

### *Lemna minor* assay

IC_50_ values and corresponding 95% confidence limits for the growth of *L. minor*, recorded after the exposure to the elutriates of the different mine soils are described in Table 3. Only elutriates of D1, D2, D3, E1, F1, F2 and F3 soils have significantly inhibited the growth of this aquatic plant species. Nevertheless, soil elutriates have displayed quite different toxicities with the lowest IC_50_ values recorded in the first samples of transects D, E and F, which were those collected near the mining and ore treatment area. The high availability of As and Cu, was probably responsible for the high toxicity of the elutriate from soil F1 and D1 to *L. minor*. The high phytotoxicity of As results from its ability to mimic phosphorus, causing negative effects in plants metabolic activity [42]. In fact, the concentration of As in the elutriate of the F1 soil was similar to the EC_50_ value reported by Duester et al. [43] for As (V) and for *L. minor* growth (82 mg L^−1^: 95%CI = 76–87). Even though we have not determined As speciation in our elutriates, this form of arsenic is expected to occur at high concentrations, since As (III) tends to oxidize to As (V) in aqueous suspensions. Copper is also a metal very toxic to *L. minor*. Teisseire et al. [44] determined an IC_50_ of 0.16 mg L^−1^, which was well below the extractable concentration of Cu found for soils D1 and E1. As far as elutriates of soils D2, D3, F2 and F3 are considered, their toxicity was probably related with aluminum because it was the metal present at highest concentration in the calcium chloride extracts. Nevertheless, Radić et al. [45] have shown the ability of *L. minor* to tolerate concentrations of Al up to 8.09 mg L^−1^ due to their great ability to up-regulate anti-oxidant defenses. Further, we cannot forget the possible differences between metal concentrations extracted with calcium chloride and those extracted with Steinberg medium that were used to produce the soil elutriates tested with *L. minor*. Complexation with organic components of the medium may have promoted a greater availability of metals to the macrophyte. Further, potential synergistic effects between all the metals in elutriates, even at lower concentrations could not be ignored. The known tolerance of *L. minor* and its ability to accumulate metals [45] has supported the suggested use for remediation purposes. However, in this study, *L. minor* was sensitive to different soil elutriates, even with low concentration of metals.

**Table 3:** IC_50_ values and corresponding 95% confidence limits for growth inhibition of *L. minor* exposed to elutriates of different soil samples collected in the Ervedosa mine area. NT stands for no toxicity.

	IC_50_
**REF**	NT
**C1**	NT
**C2**	NT
**C3**	NT
**C4**	NT
**D1**	2.77<4.28<5.79
**D2**	37.92<57.03<76.15
**D3**	60.21<87.36<114.5
**D4**	NT
**E1**	17.12<24.66<32.19
**E2**	NT
**E3**	NT
**E4**	NT
**F1**	0.19<0.22<0.25
**F2**	23.75<55.67<87.58
**F3**	31.93<45.18<58.43
**F4**	NT

More concerning in terms of risk assessment, was the inhibitory effect on the growth of *L. minor*, recorded for soil elutriates 2 and 3 of the segments D and F. These segments are those extending from the mining area to the River Tuela and parallel to the same river, respectively. The results obtained suggest that there may be a poor retention of the soil near the stream, which contributes for the mobilization of a mixture of metals to the soil aqueous phase and then to the aquatic ecosystem, with potential impact on its biological populations. This suspicion justifies a more detailed evaluation of this water stream, since a contamination, especially with As, may be occurring, with potential risks to natural communities and humans.

### *Zea mays* assay

The assay was validated, since more than 50% of the seeds have emerged in the OECD soil (control), as stated by the standard protocol [25]. After confirming this, the natural REF soil was used as control in the assay, and all the statistical comparisons were made in order to minimize the influence of soil properties in the physiological parameters evaluated. In fact no significant statistical differences were recorded between the REF and the OECD soil for almost all the parameters evaluated (except for fluorescence parameters). No seed germination was recorded in the F soil replicates, since the data available for all the other parameters are unavailable for this soil.

In terms of the parameters recommended by the ISO 11269-2 protocol [8], no significant differences were recorded between the REF and all the other soils, in the average number of emerged seeds (F = 1.38; d.f. = 55, 76; p = 0.185). The average number of emerged seeds varied between 42.5 and 80% (except for soil F1). The lack of sensitivity of this parameter to soil contamination with metals has already been reported by several authors [3], [46], [47]. Such fact results from the protection given to embryos by seed coverage. However, this fact is species and metal dependent [48]. In this study it was possible to perceive, once more, that seed germination was inhibited only when extremely high concentrations of metals/metalloids (As in particular) had the potential to mobilize to the soil aqueous phase, becoming bioavailable. This reinforces the usefulness of this parameter only to identify worst-case scenarios of contamination, and probably more important to worst-case scenarios of metals bioavailability.

**Figure 2:**
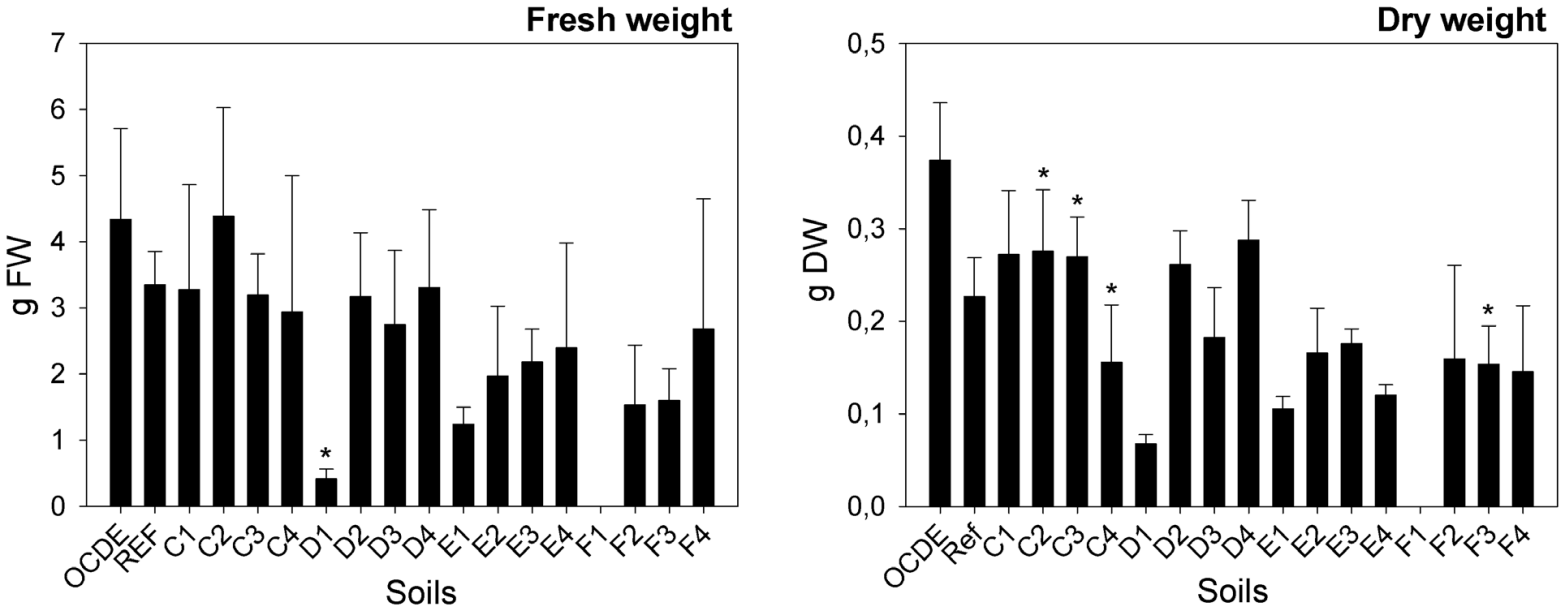
Average fresh and dry weight. Average fresh and dry weight of plants (g FW and gDW) exposed to different soils collected in Ervedosa mine area and to REF and OECD artificial soil. The error bars represent the standard deviation.

Concerning the average fresh and dry biomass above soil, significant differences among plants exposed to the different mine soils were recorded (F = 2.097; d.f. = 59,43; p = 0.029 and F = 7.722; d.f. = 55, 38, p = 0.000, respectively). A significant reduction in fresh weight was recorded only for plants exposed to soil D1 (GHT: p = 0.025) when compared with the REF soil (Figure 2). Plants from soils C4 and F3 (GHT: p = 0.017) displayed a significant lower dry weight (Figure 2). The opposite was recorded for plants exposed to soils C2 (GHT: p = 0.001) and C3 (GHT: p≤0.001), which have displayed a substantial high dry biomass when compared to plants exposed to the REF soil.

**Figure 3:**
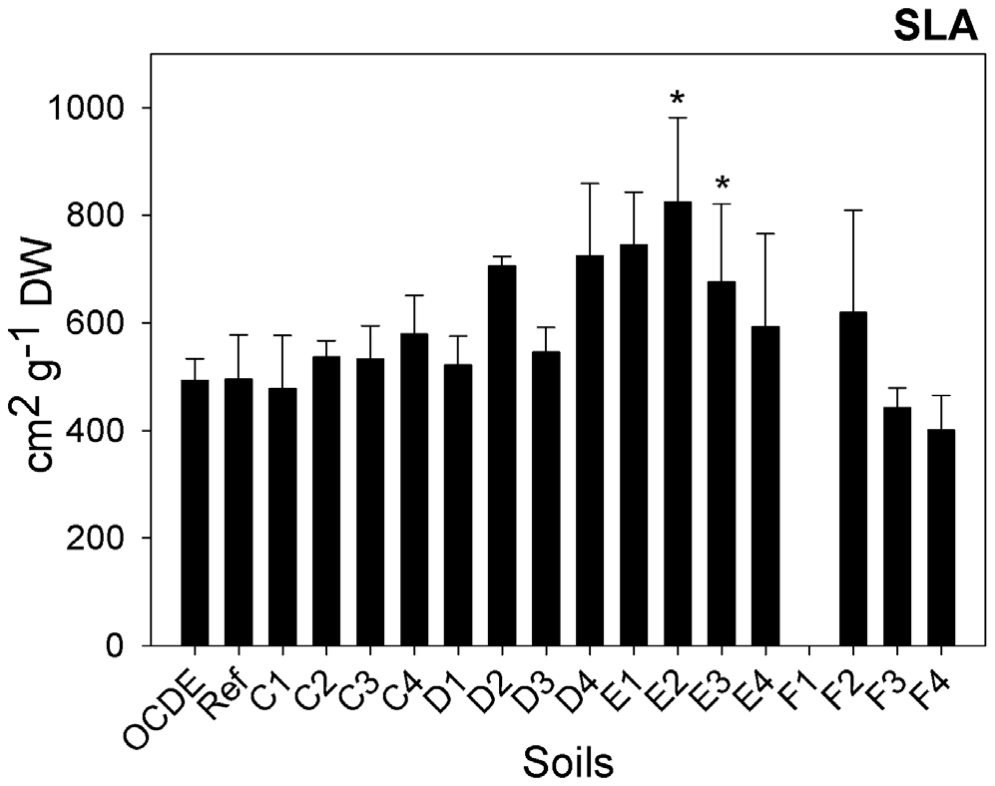
Specific leaf area. Specific leaf area (SLA) of plants (cm^2^ g^−1^ DW) exposed to different soils collected in Ervedosa mine area and to REF and OECD artificial soil. The error bars represent the standard deviation and * correspond to significant differences towards the REF soil.

**Figure 4:**
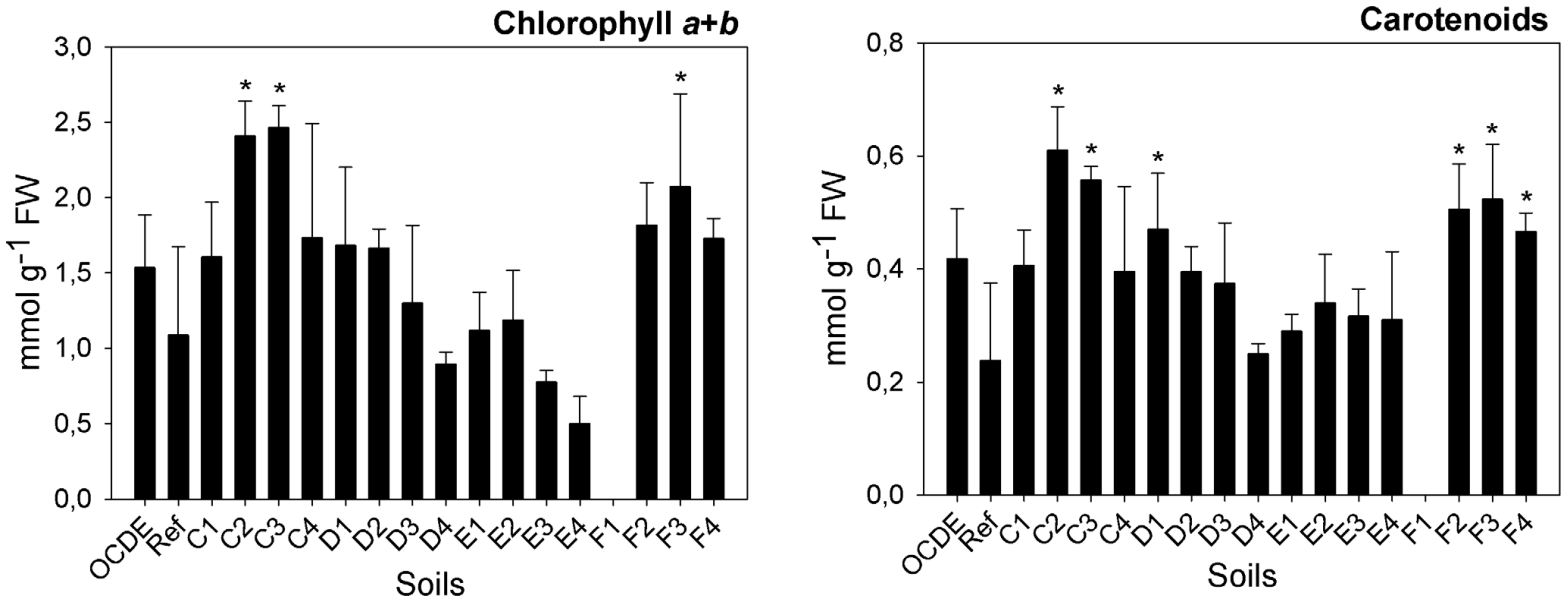
Chlorophyll *a*+*b* and carotenoids. Chlorophyll *a*+*b* (µmol g^−1^ FW) and caroptenoids in plants (mmol g^−1^ FW) exposed to different soils collected in Ervedosa mine area and to REF and OECD artificial soil. The error bars represent the standard deviation and * correspond to significant differences towards the REF soil.

**Figure 5:**
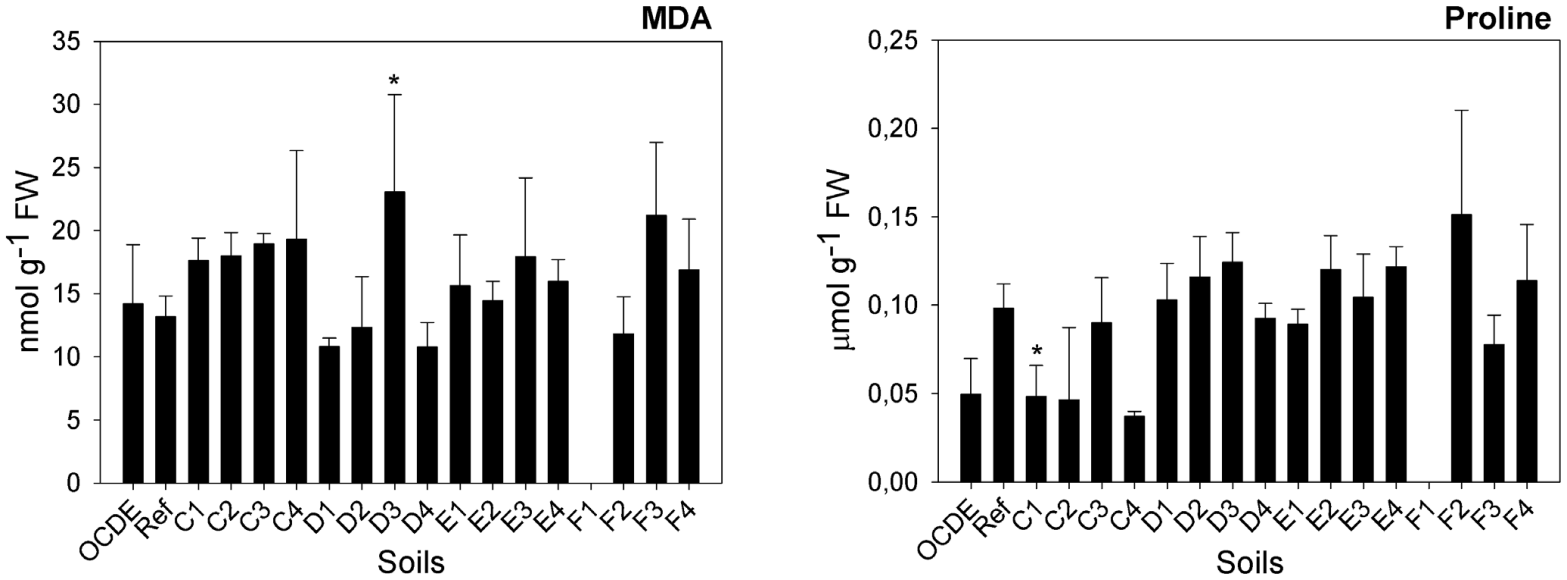
Malondialdehyde and proline content. Malondialdehyde (MDA) (nmol g^−1^ FW) and proline content in plants (µmol g^−1^ FW) exposed to different soils collected in Ervedosa mine area and to REF and OECD artificial soil. The error bars represent the standard deviation and * correspond to significant differences towards the REF soil.

As far as parameters related with plants growth and development, other than those included in the ISO protocol, are considered, significant differences in the specific leaf area (SLA) were recorded amongst plants exposed to the different mine soils (F = 3.263; d.f. = 47, 34; p = 0.003). A significant increment in this parameter was recorded for soils E2 (GHT: p≤0.001) and E3 (GHT: p = 0.028) (Figure 3). In terms of biochemical parameters, at the end of the assay, significant differences in total chlorophyll *a+b* and carotenoids contents were recorded in leaves of plants exposed to different mine soils (F = 6.576; d.f. = 50, 34; p≤0.001 and F = 5.217; d.f. = 50, 34; p≤0.001, respectively) (Figure 4). Plants from soils C2 (GHT: p = 0.002), C3 (GHT: p = 0.001) and F3 (GHT: p = 0.032) have displayed a significant higher content of chlorophylls *a+b*, while the same soils plus soils D1 (GHT: p = 0.019), F2 (GHT: p = 0.005) and F4 (GHT: p = 0.023) have induced a significant increment in the content of carotenoids of maize plants. Due to their different physical and chemical properties, metals have three different mechanisms of toxicity: production of reactive oxygen species (ROS), blocking of functional groups of enzymes and displacement of metal ions from biomolecules [16], [41]. In turn, ROS induce oxidative damage to pigments, proteins and lipids in the thylakoid membranes, compromising the overall photosynthetic activity [19]. Consequently, metals toxicity usually activates anti-oxidant defenses [49]. Carotenoids are non-enzymatic antioxidants that protect plants against photoxidation, protecting chlorophyll molecules from oxidative damages [14], [50]. Hence, the increment in the production of carotenoids content may express a response of plants to counteract the toxic effect of metals. The same occurrence was reported for other plants species, exposed to different metals (e.g. 51) or to wastes rich in metals [52]. However, in plants exposed to at least one Ervedosa mine soil (F3) such response was probably insufficient since a significant reduction in biomass still occurred. However, no significant lipid peroxidation was recorded. In fact, despite the slight increase in the MDA content in tissues of plants exposed to soils from transect C and also in some soils from transect E and F, a significant increment was observed only in plants exposed to soil D3 (Figure 5). Such observations indicate that only in these plants were the physiological mechanisms not efficient in counteracting the oxidative stress. As far as total chlorophyll contents are again considered, our results do not comply with the findings of other authors, reporting a decrease in total chlorophyll content caused by metals stress [53], [54], at least for soil F3. Plants exposed to this soil have shown a significant increment in total chlorophyll content despite the significant reduction in their dry biomass. Nevertheless, this could have been a punctual situation in which the plants have tried to adapt to metal exposure by increasing chlorophyll synthesis. Nevertheless, this is only an explanative hypothesis, requiring further confirmation.

Several studies, reporting the physiological responses of plants to metals stress, have shown that the amino acid proline usually accumulates in response to metal/metalloid (As included) exposures [21], [53], [54]. In fact proline has a central role in the ability of plants to react to abiotic stress [21], since it acts as a mediator in osmotic balance, protects macromolecules during dehydration and acts as a hydroxyl radical scavenger [14]. In this study no changes were recorded in this parameter, except for soil C1 (Figure 5). Plants exposed to this soil showed a significant reduction in their proline content (F = 3.895; d.f. = 62, 46; p≤0.001; Dunnet: p≤0.001). Although contradicting general findings, Pavlík et al. [21] have suggested that under As stress the biosynthesis of proline could be inhibited, due to a preferred utilization of glutamate, which in turns leads to the synthesis of phytochelatins. It was shown that the synthesis of phytochelatins, also called class III metallothioneins, is activated in plant cells after exposure to different metals, as part of another important detoxification mechanism [49].

**Figure 6:**
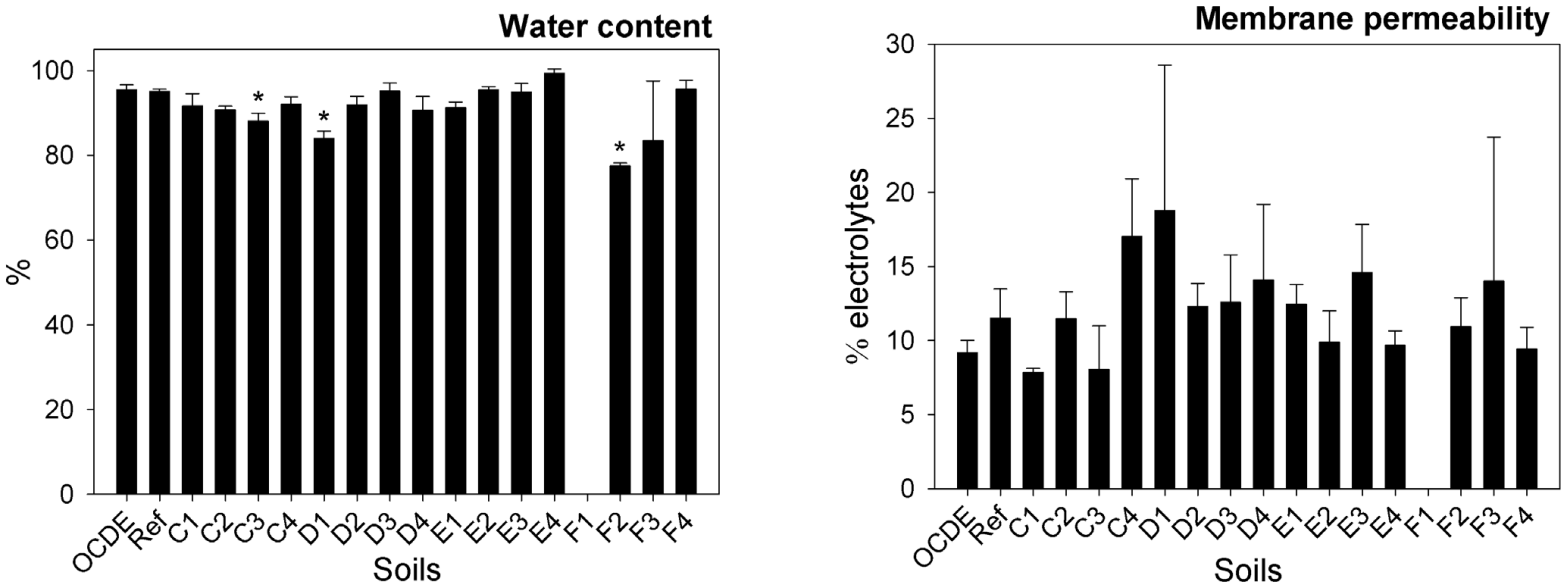
Water content and membrane permeability. Water content (%) and membrane permeability (% electrolytes) in plants exposed to different soils collected in Ervedosa mine area and to REF and OECD artificial soil. The error bars represent the standard deviation and * correspond to significant differences towards the REF soil.

Plants exposed to soils C3, D1 and F2 have also shown a significant reduction in leaf water content (F = 3.282; d.f. = 63, 47; p = 0.001; GHT: p = 0.003) (Figure 6). Since no significant differences in terms of cells membrane permeability was observed (F = 1.558; d.f. = 46, 30; p = 0.147) between plants exposed to the different mine soils (Figure 6), we can suggest that the reduction in water content in plants exposed to soil F3, was probably due to an inhibition in root growth with subsequent reduction in water uptake. Different authors [55], [56] have reported the inhibition of roots growth caused by metals/metalloids like As and Cu, in *Triticum aestivum* and *Helianthus annuus*, respectively. Nevertheless, this parameter was not assessed in this study.

**Figure 7:**
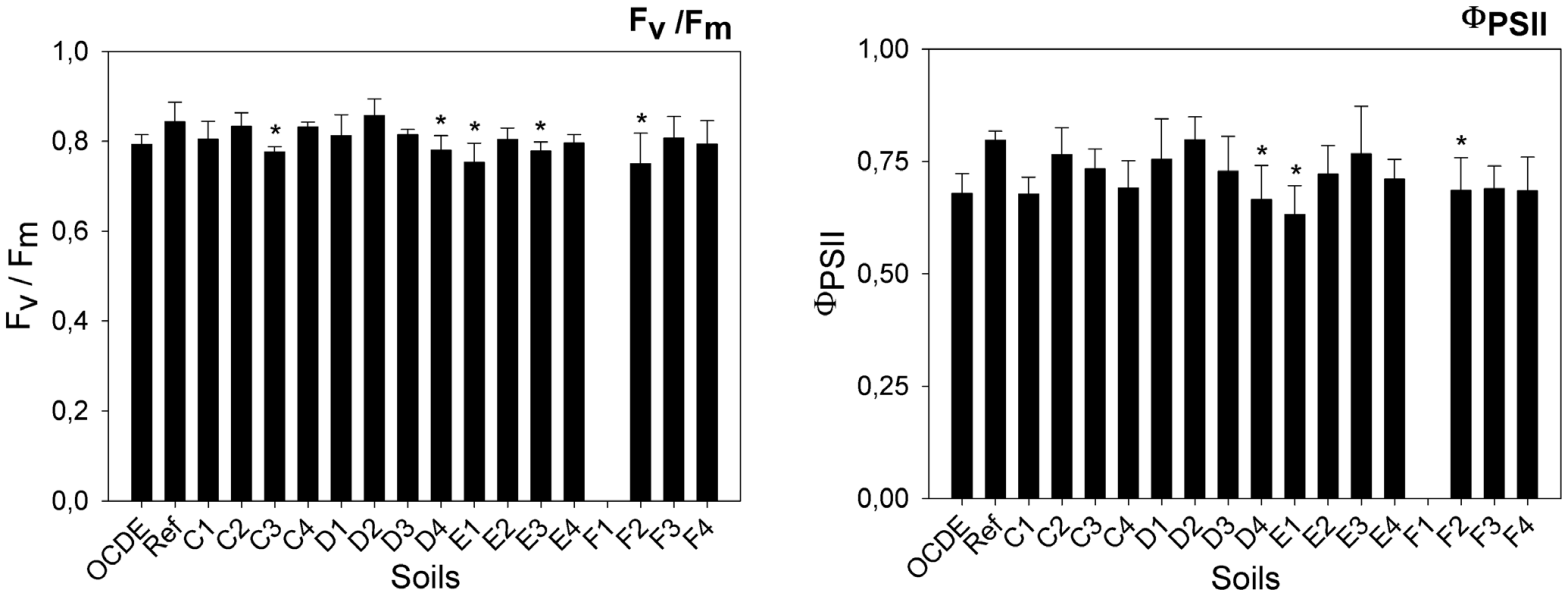
Maximum quantum yield and efficiency of photosystem II. Maximum quantum yield or the maximum photosynthetic efficiency of photosystem II (F_v_/F_m_) and efficiency of photosystem II (Φ_PSII_) in plants exposed to different soils collected in Ervedosa mine area and to REF and OECD artificial soil. The error bars represent the standard deviation and * correspond to significant differences towards the REF soil.

An efficient photosynthesis is crucial for plant survival and fitness [19], and chlorophyll fluorescence can give information about the state of the photosynthetic apparatus and, of photosystem II [20] in particular, which is considered to be the most vulnerable component. In terms of chlorophyll fluorescence parameters measured in this study, namely F_v_/F_m_ ratio (F = 5.058; d.f. = 121, 105; p≤0.001) and Φ_PSII_ (F = 4.335; d.f. = 122, 106; p≤0.001) significant differences among the plants exposed to the different mine soils were recorded for both parameters (Figure 7). The F_v_/F_m_ ratio, which measures the photochemical efficiency of photosystem II (PSII) in the dark-adapted state, was significantly reduced in plants exposed to soil C3 (GHT: p = 0.006), D4 (p = 0.009), E1 (GHT: p≤0.001), E3 (GHT: p = 0.011) and F2 (p≤0.001), when compared to the reference soil (Figure 7). In all these soils the plants have displayed average F_v_/F_m_ ratios below 0.80. According to Björkman and Demming [57] the F_v_/F_m_ ratio is almost constant for different plant species, under non-stressed conditions and, usually varies between 0.80–0.86. Values below this range suggest impairments in the photosynthetic apparatus. This possibility of damages was further reinforced for soils D4 (GHT: p≤0.001), E1 (GHT: p≤0.001), F2 (GHT: p = 0.020), which has also shown a significant reduction in Φ_PSII_ values (Figure 7). As it was demonstrated by Küpper et al. [58], different metallic cations may replace the central magnesium ion of the chlorophyll molecules, resulting in “*heavy metals substituted chlorophylls (hm-chls)*” reducing light harvesting by these molecules and, subsequently, reducing their fluorescence yields and compromising photosynthesis. Further, these authors have proved that the rate of substitution reactions varies with light intensity. At lower intensities *hm-chls* are more stable, and plants could appear vital, even when dead. Such fact could explain why a slight decrease in fluorescence parameters was recorded, at least for plants exposed to some soils, even without a concomitant reduction in the total chlorophyll content. Although the light intensity, to which plants were exposed during the assay, was within the range recommended by the standard protocol, the levels were lower than those recorded under a normal sunny day, in temperate latitudes.

The germination and early growth of plants are parameters that cannot be neglected in the evaluation of soils phytotoxicity since they integrate the overall effects of stress [46]. However, some authors suggested the evaluation of other parameters, at lower levels of organization, which may be more sensitive to the impact of chemicals, allowing both the early detection of physiological effects and the comprehension of their mechanisms of action [59], [60], [61], [62]. In this study, the key biomarkers evaluated in the *Zea mays* seedlings were parameters related with plant development, photosynthetic activity, water balance, the synthesis of secondary metabolites, oxidative stress, and detoxification mechanisms. Table 4 summarizes the results, presenting the significant effects detected for each parameter evaluated in *Z. mays* plants exposed to the different soils. As it was possible to perceive by grey columns, five additional soils (C1, D2, D4, E1, F2) induced stress on *Z. mays* with the evaluation of other plant performance parameters. Fluorescence parameters were the more sensitive and those with a greater contribution to detect false negative results in terms of phytotoxicity. In addition, elutriates of three of these soils (D2, E1 and F2) have also proved to be toxic to *L. minor*. Hence, this new evaluation of phytotoxicity contributes to increase the evidence of risks posed by these soils. A great number of phytotoxic soils conform to previsions based on comparisons of soils total metal contents with soil benchmark values.

**Table 4:** Summary of the significant effects recorded for all the parameters measured in the *Zea mays* assay, in plants exposed to the different mine soils (arrows point out for significant increments in comparison with REF plants).

		C1	C2	C3	C4		D1	D2	D3	D4		E1	E2	E3	E4		F1	F2	F3	F4
Parameters																				
Seed germination																	X			
Fresh biomass							X													
Dry biomass			X ( 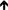 )	X ( 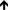 )	X		X												X	
Specific Leaf Area													X ( 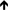 )	X ( 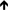 )						
Carotenoids content			X ( 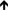 )	X ( 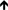 )			X ( 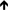 )											X ( 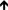 )	X ( 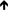 )	X ( 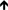 )
Chlorophylls *a*+*b*			X ( 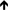 )	X ( 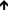 )															X ( 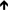 )	
MDA content								X ( 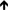 )												
Proline content		X																		
Membrane permeability (% of electrolytes)																				
Water content																			X	
F_v_/F_m_				X						X		X		X				X		
Φ_PSII_		X			X					X		X						X	X	

In summary, we can conclude that the inclusion of other physiological (chlorophyll fluorescence and/or stress oxidative parameters) in standard protocols for assays with terrestrial plants can improve their sensitivity, contributing for a more accurate evaluation of risks posed by contaminated soils. Chlorophyll fluorescence parameters, in particular, are non destructive and their measurement does not require specialized skills. However, a similar evaluation should be made, previously, for soils with different kinds of contamination.
